# Perimovement decrease of alpha/beta oscillations in the human nucleus accumbens

**DOI:** 10.1152/jn.00142.2016

**Published:** 2016-07-13

**Authors:** Max-Philipp Stenner, Stefan Dürschmid, Robb B. Rutledge, Tino Zaehle, Friedhelm C. Schmitt, Jörn Kaufmann, Jürgen Voges, Hans-Jochen Heinze, Raymond J. Dolan, Mircea Ariel Schoenfeld

**Affiliations:** ^1^Department of Neurology, Otto von Guericke University, Magdeburg, Germany;; ^2^Department of Behavioral Neurology, Leibniz Institute for Neurobiology, Magdeburg, Germany;; ^3^Wellcome Trust Centre for Neuroimaging, University College London, London, United Kingdom;; ^4^Max Planck University College London Centre for Computational Psychiatry and Ageing Research, London, United Kingdom; and; ^5^Department of Stereotactic Neurosurgery, Otto von Guericke University, Magdeburg, Germany

**Keywords:** nucleus accumbens, beta oscillations, action preparation, deep brain stimulation

## Abstract

The human nucleus accumbens is thought to play an important role in guiding future action selection via an evaluation of current action outcomes. Here we provide electrophysiological evidence for a more direct, i.e., online, role during action preparation. We recorded local field potentials from the nucleus accumbens in patients with epilepsy undergoing surgery for deep brain stimulation. We found a consistent decrease in the power of alpha/beta oscillations (10–30 Hz) before and around the time of movements. This perimovement alpha/beta desynchronization was observed in seven of eight patients and was present both before instructed movements in a serial reaction time task as well as before self-paced, deliberate choices in a decision making task. A similar beta decrease over sensorimotor cortex and in the subthalamic nucleus has been directly related to movement preparation and execution. Our results support the idea of a direct role of the human nucleus accumbens in action preparation and execution.

## Abstract (precis)

*The present work clarifies how the nucleus accumbens contributes to action. This region is often assumed to influence behavior “off-line” by evaluating outcomes. Studying rare recordings of local field potentials from the human nucleus accumbens, we observe a perimovement decrease of alpha and beta oscillations in seven of eight individuals, a signal that, in the motor system, is directly related to action preparation. Our results support the idea of an online role of this region for imminent action*.

## NEW & NOTEWORTHY

*The present work clarifies how the nucleus accumbens contributes to action. This region is often assumed to influence behavior “off-line” by evaluating outcomes. Studying rare recordings of local field potentials from the human nucleus accumbens, we observe a perimovement decrease of alpha and beta oscillations in seven of eight individuals, a signal that, in the motor system, is directly related to action preparation. Our results support the idea of an online role of this region for imminent action*.

the nucleus accumbens (NAcc) is thought to play an important role in decision making for action (Floresco 2015; Goto and Grace 2008; Mogenson et al. 1980). Pharmacological and lesion studies in rodents have shown that the NAcc is involved in instrumental learning and performance (Atallah et al. 2007; Corbit et al. 2001), set shifting (Floresco et al. 2006), control of impulsive behavior (Cardinal et al. 2001), and suppression of unconditioned behaviors (Krause et al. 2010). Therapeutic effects of deep brain stimulation (DBS) of the NAcc in neuropsychiatric disorders, in particular improvement of compulsive behaviors in treatment-refractory obsessive-compulsive disorder (Denys et al. 2010; van Westen et al. 2015), suggest an important role of the NAcc also in complex aspects of human behavior.

The NAcc is often assumed to influence behavior via an evaluation of action outcomes. Supporting this view, functional imaging studies and single-unit recordings in humans describe signals in the NAcc consistent with reward prediction errors (RPEs), the difference between actual and expected rewards (Abler et al. 2006; Patel et al. 2012; Rutledge et al. 2010; but see Stenner et al. 2015b). In reinforcement learning models, RPEs are essential teaching signals for instrumental control (e.g., Pessiglione et al. 2006). RPE signals in the NAcc have been taken as evidence that the ventral striatum, which includes the NAcc, functions as a “critic” (Sutton and Barto 1998) by learning to predict reward, while an “actor” located elsewhere in the brain performs action selection (Montague et al. 2004; O'Doherty et al. 2004). In this framework, the NAcc fulfils an indirect, “off-line,” behavioral function at a time when no action is being prepared rather than contributing directly to the ongoing selection of imminent actions.

However, there is also evidence of a more direct, “online” (Pennartz et al. 2009) role of the NAcc in action selection. Recording from neurons in the rat NAcc during a spatial navigation task, van der Meer and Redish (2009a) observed that ensemble firing patterns at a decision point and prior to error correction resembled reward-induced patterns, a finding the authors interpreted as an intrinsically driven expectation signal during decision making. In a recent study of local field potentials (LFPs) from the human NAcc we found a cortical drive of delta oscillations prior to choices in an economic decision making task (Stenner et al. 2015a), in line with findings of a similar coupling during instrumental behavior in rodents (Gruber et al. 2009). Finally, Patel et al. (2012) observed that single-unit activity in the human NAcc predicted the size of a subsequent monetary bet in a computerized card game. Together, these preaction signals are consistent with a direct contribution of the NAcc to ongoing preparation for imminent action, beyond its putative function in evaluating action outcomes.

In the cortex, preparing for imminent motor action is typically accompanied by distinct signal variations in the scalp EEG. Specifically, the power of alpha and beta oscillations decreases over sensorimotor cortex prior to and around movement time, a phenomenon referred to as event-related desynchronization (ERD; Pfurtscheller et al. 2003). A similar perimovement ERD of beta oscillations has been described in motor-related subcortical areas, specifically the subthalamic nucleus (Cassidy et al. 2002; Kühn et al. 2004; Litvak et al. 2012). Sensorimotor and subthalamic beta oscillations are considered to support posture maintenance and inhibit movement (Engel and Fries 2010; Gilbertson et al. 2005; Joundi et al. 2012; Kühn et al. 2004; Swann et al. 2009). Conversely, their decrease during movement is thought to reflect processes directly related to the preparation of imminent motor actions across a cortico-subcortical network.

In light of these results, we expected to find a similar perimovement beta-ERD in the NAcc. We recorded LFPs from the NAcc of nine epilepsy patients via DBS electrodes. In two different tasks, patients moved their fingers in response to visual cues or to make deliberate economic choices. The presence of a beta-ERD within the NAcc before these movements would indicate a direct, i.e., online, role in action preparation or execution.

## METHODS

### Patients

We recruited nine patients with pharmacoresistant partial epilepsy (mean ± SD age: 40.6 ± 8.3 yr; 4 women, 5 men; see Table 1 for details), who underwent surgery for implantation of DBS electrodes. One patient (*P9*) was excluded because of a low signal-to-noise ratio of LFPs from the NAcc in both hemispheres (see *Recording and Analysis of Local Field Potentials*; see also Stenner et al. 2015b). All patients participated in in-house protocols to study the safety and anti-ictal efficacy of NAcc DBS (Kowski et al. 2015; Schmitt et al. 2014). Both this clinical trial and the experiments reported here were approved by the institutional ethics review board of the University of Magdeburg (registration no. 03/08). All patients gave written informed consent.

**Table 1.** Clinical data

ID	Sex/Age/Disease Duration, yr	Epilepsy Syndrome	Etiology	Seizure Lateralization	Seizure Onset	AEDs
*P1*	F/52/19	Multifocal	Cryptogenic	Bilateral	Mesio-temporal	LCM 400 mg
						LTG 200 mg
*P2*	M/35/9	Focal	Right temporal encephalocele	Right	Temporal	LEV 2,000 mg
						ESL 1,200 mg
*P3*	F/28/12	Multifocal	Cryptogenic	Bilateral	Temporal	LTG 200 mg
						LCM 400 mg
*P4*	M/40/31	Focal	Left hippocampal sclerosis†	Left	Temporal	LTG 400 mg
						LCM 400 mg
*P5*	M/32/31	Multifocal	Genetic (SCNA1)	Left, possibly bilateral	Bifrontal and left medio-temporal	OXC 900 mg, clobazam 5 mg
						STP 4,500 mg
*P6*	F/52/17	Focal	Cryptogenic	Left	Temporal	LTG 250 mg
						LCM 400 mg
*P7*	F/44/14	Multifocal	Right hippocampal sclerosis*	Bilateral	Temporal	CBZ 1,200 mg
*P8*	M/39/9	Multifocal	Cryptogenic	Bilateral	Bifrontal	LCM 400 mg
						ZNS 400 mg
*P9*	M/43/38	Left temporal	Posttraumatic lesion	Left	Temporal	LCM 400 mg
						LEV 3,000 mg

AED, antiepileptic drug; LCM, lacosamide; LTG, lamotrigine; LEV, levetiracetam; ESL, eslicarbazepine acetate; OXC, oxcarbazepine; STP, stiripentol; CBZ, carbamazepine; ZNS, zonisamide.

* Underwent right temporal lobe resection 3 yr before DBS surgery;

† underwent left temporal lobe resection 9 yr before DBS surgery.

### Electrode Implantation

**Fig. 1.**
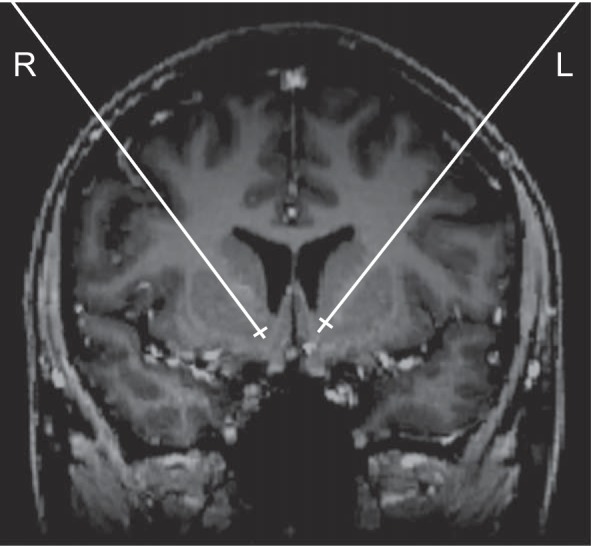
Exemplary presurgical MRI in 1 patient (*P6*) showing a projection of the planned placement of electrodes in bilateral NAcc onto a coronal MRI slice through the striatum.

Each patient underwent stereotactically guided implantation of four quadripolar electrodes, one for each NAcc and anterior thalamus. Presurgical planning of electrode placement and surgical procedures were conducted as previously described in detail (Voges et al. 2002; Zaehle et al. 2013). In brief, standardized coordinates for the NAcc were adjusted to each individual's presurgical MRI. Electrodes were placed 2–2.5 mm lateral to the vertical limb of Broca's band, such that the two most ventral contacts of each quadripolar electrode were placed inside the NAcc, covering parts equivalent to the NAcc shell and core regions in rodents (Fig. 1). The two dorsal contacts were located in the NAcc, adjacent striatum (ventral pallidum, fundus caudati), or the anterior limb of the internal capsule. After surgery, electrode leads were externalized for 5–6 days to allow for clinical test stimulation with varying parameters.

### Procedure and Tasks

We report reanalyses of two separate data sets, recorded during a serial reaction time task and an economic decision making task, respectively (Dürschmid et al. 2013; Stenner et al. 2015a, 2015b). Both data sets were collected between the second and fifth day after electrode implantation. For each task data were collected from five of the included patients, two of whom completed both tasks. Stimuli were controlled via custom software in MATLAB (MathWorks) and presented on a laptop computer.

#### Serial reaction time task.

**Fig. 2.**
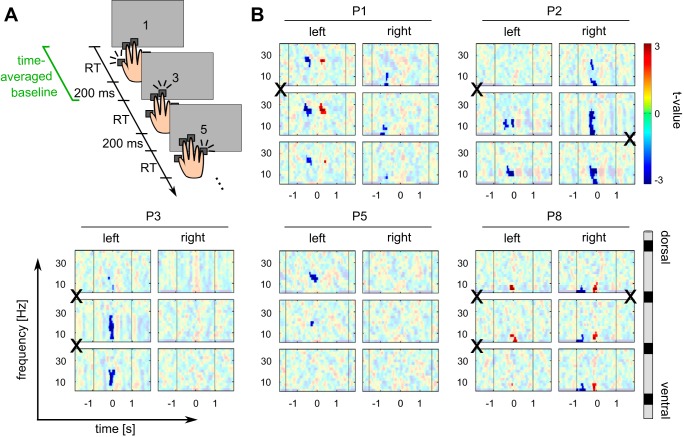
Perimovement decrease of the power of beta oscillations in the serial reaction time task. *A*: schematic of 3 consecutive trials, including timing of trial events. Cues (numbers on the screen, indicating which key to press) could be presented in a fixed, repetitive order (“1,” “3,” “5,” “3,” “2,” “3”; fixed sequence condition) or in random order (random sequence condition) or they could be replaced by a fixation cross (uninstructed choice condition, free choice). Conditions were blocked. Baseline power was computed for each trial, channel, and frequency bin separately by averaging power from the onset of the visual cue to the onset of the subsequent cue (i.e., the beginning of the subsequent trial; this interval is depicted in green). RT, reaction time. *B*: time- and frequency-resolved power spectra around the time of a key press (*time 0*) for each of the 5 patients who completed the serial reaction time task (*P1*, *P2*, *P3*, *P5*, and *P8*) for each of the 3 DBS channels in each hemisphere. For each patient, all 3 bipolar channels (rows) are shown for the left and right hemispheres (columns), from dorsal (*top*) to ventral (*bottom*) channels. Color codes for *t*-values (perimovement power vs. time-averaged power, see methods). Opaque areas correspond to *P* ≥ 0.05. X indicates polarity reversals in the beta-frequency band. Black vertical lines to the *left* and *right* of *time 0* indicate median response onset in the preceding and subsequent trials, respectively. A premovement decrease of the power of beta oscillations is observed in at least 1 DBS channel in all patients except *P8*.

In each trial of the serial reaction time task (Dürschmid et al. 2013; Nissen and Bullemer 1987), patients pressed one of four keys on the laptop keyboard with a finger of their right hand as instructed by a number presented on the laptop monitor. Specifically, patients were instructed to press the space bar, “j,” “k,” or “;” (German keyboard) as quickly as possible with the thumb or index, middle, or little finger of their right hand, respectively, in response to the numbers “1,” “2,” “3,” and “5,” respectively, presented at fixation at the beginning of each trial (height 2 cm; Fig. 2*A*).

There were three different conditions, presented in a fixed order. In the first condition, which consisted of three blocks of 60 trials each, numbers were presented in a fixed, repetitive sequence (“1” → “3” → “5” → “2” → “3” → “2”). In the second condition, numbers were presented in a random sequence (2 blocks of 60 trials each). In the third condition (1 block of 60 trials), numbers were replaced by a fixation cross and patients were free to choose which key to press (uninstructed choice condition). Each trial started 200 ms after the preceding key release. During this interval, stimuli in the fixed and random sequence conditions remained on the screen, while the fixation cross in the uninstructed condition disappeared to mark the interval between trials.

#### Economic decision making task.

**Fig. 3.**
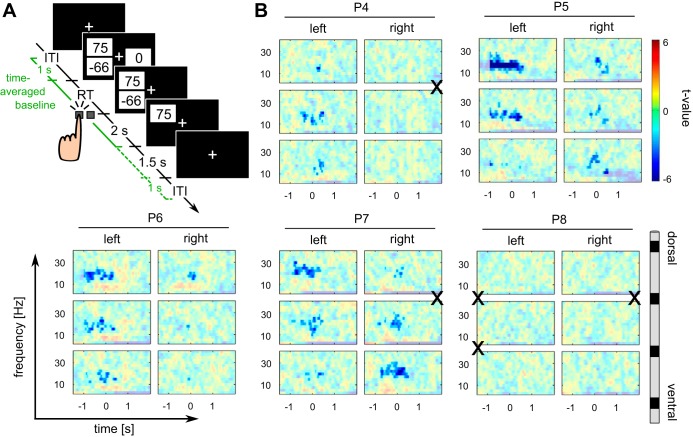
Perimovement decrease of the power of beta oscillations in the economic decision making task. *A*: schematic of 1 trial in which the gamble option is chosen, including timing of trial events. The green time intervals (solid and dashed) represent the 2 alternative time periods over which power was averaged to obtain baselines for each trial, channel, and frequency bin. These periods lasted from 1 s before the onset of the options on the screen to the onset of the outcome (solid green line) or until 1 s after the offset of the outcome (solid and dashed lines together). The intertrial interval (ITI) varied between 1.5 and 2 s (randomly drawn from a uniform distribution). RT, response time. *B*: time- and frequency-resolved power spectra around the time of a key press (*time 0*) for each patient (*P4*, *P5*, *P6*, *P7*, and *P8*) for each of the 3 DBS channels in each hemisphere. For each patient, all 3 bipolar channels (rows) are shown for the left and right hemispheres (columns), from dorsal (*top*) to ventral (*bottom*) channels. Color codes for *t*-values (perimovement power vs. time-averaged power, see methods). Opaque areas correspond to *P* ≥ 0.05. X indicates polarity reversals in the beta-frequency band. A perimovement decrease of the power of beta oscillations is observed in at least 1 DBS channel in all patients except P8.

In each of 200 trials of the economic decision making task (Rutledge et al. 2014; Stenner et al. 2015b), patients decided whether to accept or reject a monetary gamble offer, where the gamble varied from trial to trial. If accepted, a gamble resulted in a monetary gain or loss with equal probabilities. To reject a gamble, patients instead chose a safe option worth 0 euros. At the beginning of each trial, the potential win and loss amounts of the gamble option and the safe option were represented by three numbers on the screen (Fig. 3*A*). Two of the three numbers were presented on one side of the screen (left or right, counterbalanced across trials) and corresponded to the possible gain and loss of the current gamble offer. The third number, presented on the opposite side of the screen, was always 0 and represented the safe option. Patients chose the option presented on the left or right by pressing the left “Ctrl” or right “Enter” key with their left or right hand, respectively, without any time limit. If a gamble option was chosen, its outcome was randomly determined by the computer and displayed for 1.5 s after a 2-s delay period. This was followed by an intertrial interval of 1.5–2 s (drawn from a uniform distribution).

Potential gamble outcomes ranged from +25 to +100 euro cents for wins and from −5 to −200 euro cents for losses. The outcome of each trial counted for real money. Subjects were endowed with 15 euros at the start of the experiment and, in addition to this endowment, earned an average of 15.18 euros (range 3.73–27.38 euros), paid out at the end of the experiment.

### Recording and Analysis of Local Field Potentials

LFPs were recorded from four platinum-iridium contacts (1.5 mm wide, 1.5 mm apart) on each of the four DBS electrodes (bilateral NAcc and anterior thalamic nucleus) and from three or four surface EEG electrodes (*patients P1–P8*). We report data from the bilateral NAcc, for which the study was designed. LFPs were digitized at a sampling rate of 512 Hz (Walter Graphtek system; for *patient P9*, Brain Vision software was used at a sampling rate of 2,500 Hz). Following previous work (Litvak et al. 2012), all analyses are based on a bipolar montage in which each contact is referenced to its dorsal neighbor, resulting in three channels per hemisphere. This maximizes spatial specificity for the NAcc by minimizing volume conduction effects from distant sources. Events of interest (onset of visual stimuli, key presses and releases) were recorded simultaneously to LFPs via triggers sent through MATLAB. During recording, a high-pass filter of 0.19 Hz and a low-pass filter of 240 Hz were applied to LFPs.

LFPs were analyzed with FieldTrip (Oostenveld et al. 2011) and MATLAB (versions R2012b and R2009b; MathWorks). Data recorded during the serial reaction time task were divided into epochs from 2.2 s before to 2.2 s after each key press. Rare trials in which two keys had been pressed were excluded. The numbers of trials available for analysis were as follows (all conditions/fixed sequence/random sequence/uninstructed choice): 357/177/120/60 (*P1*), 354/177/119/58 (*P2*), 358/178/120/60 (*P3*), 355/179/117/59 (*P5*), and 355/177/119/59 (*P8*). Data recorded during the economic decision making task were epoched from 1.5 s before option onset to 5 s after the choice (i.e., 3 s after outcome onset in trials in which patients accepted the gamble). Line noise was removed from both data sets with a narrow-band, fourth-order, two-pass Butterworth filter (48.5–51.5 Hz and harmonics up to 250 Hz). Data from the economic decision making task, which included longer interstimulus intervals and response times than the serial reaction time task, underwent artifact rejection with standard options in FieldTrip. Specifically, trials were rejected if the maximum amplitude variance across all available channels exceeded a threshold. This resulted, on average, in a rejection of 19% of all trials. The numbers of trials available for analysis of the economic decision making task were 178 (*P4*), 165 (*P5*), 186 (*P6*), 143 (*P7*), 132 (*P8*), and 168 (*P9*).

Inspection of artifact-free data revealed a low signal-to-noise ratio of NAcc recordings from *patient P9*. As in previous work (Cohen et al. 2009a, 2009b), we expected that the presentation of the two options on the screen, as well as the presentation of the gamble outcome, would each result in an evoked response within the first 800 ms. While we indeed found at least one significant (*P* < 0.05) positive or negative peak in both of these time windows in *patients P4–P8*, data from none of the six DBS channels from *patient P9* differed significantly from zero in response to either event (*P* > 0.1; corrected for multiple comparisons across all time bins between 0 and 800 ms, separately for both time windows; cluster-based permutation test, see below). This indicated a low signal-to-noise ratio. *Patient P9* was therefore excluded from all analyses (see also Stenner et al. 2015b).

For spectral analysis, Fourier-transformed data segments sampled every 25 ms were multiplied with a Fourier-transformed Hanning taper (window length 400 ms). Spectral power was computed as the squared absolute of the Fourier transform. Because we were primarily interested in oscillations in the alpha and beta frequency range, we focused on frequencies between 2.5 and 40 Hz, analyzed in steps of 2.5 Hz.

To test for a time-dependent modulation of spectral power, specifically a decrease of beta power around the onset of a motor response, we compared epoched data to time-averaged power computed for each trial, channel, and frequency bin separately. For the serial reaction time task, this baseline was computed by taking the mean power across all time bins from the onset of a cue on the screen to the presentation of the next cue, i.e., the start of the following trial (Fig. 2*A*). For the economic decision making task the baseline was computed from 1 s before the onset of the options on the screen to 1 s after the offset of the outcome (Fig. 3*A*). To exclude the possibility that perimovement power deviations from baseline in the decision making task were due to outcome-induced power changes that entered the computation of baseline power, a control baseline period from 1 s before the onsets of options to the onset of the outcome was used (Fig. 3*A*).

To test for effects of laterality of movement (contralateral vs. ipsilateral to the NAcc), we followed a double-subtraction approach typically used to compute lateralized motor cortical signals, such as the lateralized readiness potential (Eimer 1998). To this end, we first subtracted, for each trial, time-frequency power recorded from the right vs. left DBS electrode. We then tested whether this interhemispheric power difference was significantly larger or smaller for trials with a left-hand vs. a right-hand movement.

For evidence of a local origin of alpha/beta oscillations recorded from DBS electrodes, we tested for polarity reversals between adjacent bipolar channels (Brown et al. 2001; Kühn et al. 2004) by cross-correlation (“xcorr.m” in MATLAB) of band-pass filtered data (10–30 Hz). Cross-correlograms were computed for single epochs and then averaged across trials. Negative peaks at a lag of ±6 ms were considered polarity reversals (Doyle et al. 2005).

While the relatively small sample sizes in our study limit statistical group-level inference, our data sets offer an excellent opportunity to study the consistency of task-induced modulations across five individual cases in each of the two tasks (see also Stenner et al. 2015a, 2015b). To this end, we used a standard nonparametric permutation test across trials, implemented in FieldTrip, which corrects for multiple comparisons across time bins, frequency bins, and channels. First, a *t*-statistic (dependent-samples *t*-test) was computed for a given contrast of interest (perimovement modulation vs. time-averaged baseline) for each time and frequency bin in each channel separately. Adjacent time bins and frequency bins and neighboring channels whose *t*-statistic exceeded a threshold (*P* < 0.05, 2-sided) were then clustered. For each cluster, a cluster-level statistic was obtained by summing *t*-values across its elements. The null hypothesis was rejected if this cluster-level statistic exceeded a critical value estimated from a distribution obtained by at least 500 random data permutations. To this end, the perimovement time-frequency spectrum and the time-averaged baseline spectrum were randomly assigned the two labels (“perimovement,” “baseline”) independently for each trial. For each permutation, a *t*-statistic was recomputed for each time/frequency bin in each channel. Adjacent bins whose *t*-statistic exceeded a two-sided threshold of *P* < 0.05 were then clustered, and all *t*-values in a given cluster were summed to obtain a cluster-level statistic. The maximum (positive and negative) cluster-level statistic from each permutation was used to build a nonparametric distribution, reflecting the null hypothesis of no difference between perimovement spectra and baseline spectra. A *P* value was defined for each positive and negative cluster in the actual data as the proportion of random permutations with a higher or lower maximum cluster-level statistic, respectively. Clusters in the actual data for which this proportion exceeded a threshold of *P* < 0.05 (2 sided) were considered significant.

Since we expected a decrease of beta power before and around the onset of a motor response, we tested data from 1 s before to 500 ms after each response. For the serial reaction time task, all frequencies between 2.5 and 40 Hz were tested. Having confirmed a modulation of beta power in the serial reaction time task (Fig. 2*B*), we focused statistical testing of data from the economic decision making task on frequencies between 10 and 30 Hz.

## RESULTS

We tested whether a characteristic movement-related signal consistently observed in scalp EEG electrodes over sensorimotor cortex, namely, a decrease of beta power before and around the time of movement (Pfurtscheller et al. 2003), is also evident in LFPs recorded from the NAcc. The role of this region in action is often related to an off-line function in outcome evaluation, which would not be expected to generate perimovement signals. We first examined LFPs during simple, cued finger movements (see *Beta-ERD in NAcc During Instructed Movements*) and then turned to uninstructed, self-paced actions resulting from deliberate, value-based decision making (see *Beta-ERD in NAcc During Deliberate Choices*). Each of the two tasks was completed by five patients.

### Beta-ERD in NAcc During Instructed Movements

Across conditions, patients performed the serial reaction time task with a mean median response time of 804 ms (range 613-1,124 ms) and with a mean error rate of 3.55% (range 1.35–8.7%; computed across fixed and random sequence conditions). There was a significant condition effect on response times in all five patients (all *P* < 0.001, Kruskal-Wallis test). Post hoc pairwise comparisons showed that response times were significantly faster in the uninstructed choice condition than in the instructed (fixed and random sequence) conditions in all patients [all *P* < 0.001, Mann-Whitney *U*-test; mean median response times: 842 ms (fixed sequence), 838 ms (random sequence), and 397 ms (uninstructed choice condition)]. In *patient P8*, response times were also significantly faster in the fixed sequence condition compared with the random sequence condition (*P* < 0.01).

**Fig. 4.**
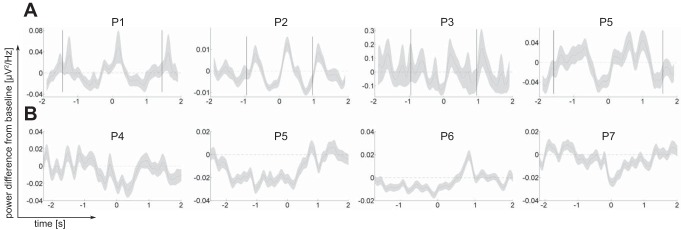
Perimovement time course of alpha/beta power in the serial reaction time task (*A*) and the economic decision making task (*B*). For each patient (*P1–P7*), baseline-corrected power (in μV^2^/Hz) was averaged across channels and frequency bins between 10 and 30 Hz that showed a significant difference from baseline for that patient (Figs. 2 and 3). Time courses are aligned to responses (*time 0*). For the serial reaction time task, black vertical lines to the *left* and *right* of *time 0* mark median response onset in the preceding and subsequent trials, respectively.

Four of the five patients who completed the serial reaction time task (*P1*, *P2*, *P3*, and *P5*) showed a significant premovement decrease of beta and alpha power in DBS channels of at least one hemisphere (Fig. 2*B* and Fig. 4*A*; *P* < 0.05, 2-sided test, corrected for multiple comparisons across all time bins between 1 s before and 500 ms after the response, all frequency bins between 2.5 and 40 Hz, and all 6 DBS channels). In three of these patients, contacts placed in the NAcc showed a beta and alpha power decrease (*P1*, *P2*, and *P3*). Latencies and frequencies of significant clusters varied slightly across individuals, with clusters extending to theta frequencies in two patients (*P2* and *P3*). On average across the four patients who showed a significant alpha/beta decrease (*P1*, *P2*, *P3*, and *P5*), significant clusters extended from −425 ms (range −675 to −225 ms) to −37 ms (range −300 to 100 ms) relative to the key press and from 10 Hz (range 2.5 to 20 Hz) to 25 Hz (range 20 to 30 Hz). In addition, one patient (*P1*) showed a significant beta power rebound after the response [similar rebounds were observed in *patients P2*, *P3*, and *P5* (see Fig. 2*B*) but did not reach significance in the permutation test].

Three of the patients who completed the serial reaction time task (*P2*, *P3*, and *P5*) showed a significant effect of condition on the premovement alpha/beta decrease in DBS channels (the power difference from baseline averaged across the clusters shown in Fig. 2*B*; *P* < 0.05, Kruskal-Wallis test). In all three, post hoc pairwise comparisons revealed that spectral power across the clusters in Fig. 2*B* decreased significantly more in the random sequence condition than in the uninstructed choice condition and in *patient P3* the fixed sequence condition. In two patients (*P2* and *P5*), there was also a significantly stronger decrease in the fixed sequence condition compared with the uninstructed choice condition.

### Beta-ERD in NAcc During Deliberate Choices

A detailed analysis of behavioral data from the economic decision making task, including computational modeling, is presented in Stenner et al. (2015b). On average, patients chose between the two options within 2.17 s (range of median response times 1.46–3.04 s). Overall, they accepted gamble offers more often than rejecting them (mean ± SD: 66 ± 13% of trials; range 46–82%), particularly when gambles had a positive expected value (calculated as the mean of the 2 possible outcomes of a gamble) compared with gambles with a negative expected value (chosen, on average, in 73 ± 12% of positive expected value trials vs. 42 ± 28% of negative expected value trials).

As in the serial reaction time task, four of the five patients who completed the economic decision making task (*P4–P7*) showed a significant decrease of the power of alpha and beta oscillations before and around the key press (Fig. 3*B* and Fig. 4*B*; *P* < 0.05, 2-sided test, corrected for multiple comparisons across all time bins between 1 s before and 500 ms after the response, all frequency bins between 10 and 30 Hz, and all 6 DBS channels). No significant modulation around the time of the key press was observed in *patient P8*, who did not show an alpha/beta decrease in the serial reaction time task, either (Fig. 2*B*). A control analysis in which baseline power was computed across a time window before outcome onset (Fig. 3*A*) showed qualitatively similar results (a significant perimovement decrease of the power of alpha and beta oscillations in *patients P4–P7*). Compared with the serial reaction time task, in which response times were shorter, significant clusters in the decision making task started earlier, as early as at least 1 s before the key press, and persisted for longer, for at least 500 ms after the key press (the first and last time bins of our time window of interest). There was no difference in the degree of alpha-/beta-ERD between trials in which patients chose to accept vs. reject the gamble offer (all *P* > 0.07).

### Laterality of Beta-ERD

In each trial of the decision making task, patients responded with either their right or left hand, depending on their choice (to accept or reject a gamble offer) and the side of the computer screen each option was presented on (right or left). To test for a laterality effect (contralateral vs. ipsilateral to the response hand), we subtracted LFPs from DBS channels of the left vs. right hemisphere and compared these difference signals between trials with a left-hand vs. a right-hand movement. We found no significant difference in any of the five patients (all *P* > 0.1, time of interest: −1 to +0.5 s after the response, frequency of interest: 10–30 Hz).

### Focal Source of Beta Oscillations

As evidence of a local origin (Brown et al. 2001; Doyle et al. 2005; Kühn et al. 2004) of beta oscillations recorded from DBS electrodes, polarity reversals between adjacent bipolar channels were observed in six of the eight patients who were included (Fig. 2*B* and Fig. 3*B*). In four of these (*patients P1*, *P2*, *P3*, and *P7*), adjacent channels with a polarity reversal also showed a significant perimovement decrease of beta power.

As evidence of a ventral source of alpha/beta oscillations, baseline power averaged across frequencies between 10 and 30 Hz that showed a significant perimovement effect was significantly larger in the most ventral compared with the most dorsal channel in 10 of 14 electrodes (1 for each hemisphere in *patients P1–P7*; Wilcoxon signed-rank test, *P* < 0.001).

## DISCUSSION

We observed a decrease in the power of beta oscillations in LFPs recorded from DBS electrodes placed in the human NAcc before and around the time of execution of simple finger movements. This decrease was present both for cued finger movements and for deliberate, self-paced choices. These results imply a more direct involvement of the human NAcc in action preparation than typically assumed. We suggest that this signal plays a functional role similar to its counterparts at sensorimotor cortical and subthalamic sites, signals considered crucial for balancing action initiation vs. inhibition.

### Functions of Cortical and Subcortical Beta Oscillations

Cortical and subcortical beta oscillations are typically studied in relation to motor control. Many studies converge on the idea that synchronous beta oscillations are involved in regulating posture maintenance vs. movement initiation. Gilbertson et al. (2005) found that voluntary finger movements are slowed during spontaneous increases in corticospinal beta synchrony, while posture-stabilizing responses to passive stretch are enhanced. In support of the idea that beta oscillations are involved in movement inhibition, beta power over the right inferior frontal gyrus (Swann et al. 2009) and within the subthalamic nucleus (Brittain et al. 2012; Kühn et al. 2004) increases while a motor response is being stopped. Direct evidence for a role of beta oscillations in movement inhibition comes from studies using transcranial electric stimulation to enhance synchronous beta oscillations, which slows movements (Joundi et al. 2012; Pogosyan et al. 2009).

On the basis of these findings, it has been hypothesized that synchronous beta oscillations indicate or promote maintenance of the “status quo” (Engel and Fries 2010) and confine novel in favor of incumbent processes (Brittain and Brown 2014). Conversely, a decrease of local and interarea beta synchrony, as observed before and during movement, has been proposed to “liberate circuits” (Brittain and Brown 2014), facilitate processing in smaller-scale networks, and increase coding capacity. The idea of increased coding capacity with decreased beta synchrony is consistent with the observation that the degree of beta-ERD over sensorimotor cortex in response to an imperative Go cue depends on the extent to which that cue conveys new information about an imminent movement. Specifically, when movement direction is fully instructed by an earlier warning cue, there is little additional beta-ERD following the onset of a subsequent Go cue. In contrast, beta power decreases more strongly when the Go cue carries previously unavailable information about movement direction (Tzagarakis et al. 2010). Here, in our serial reaction time task, we observe a similar scaling of perimovement beta-ERD with the amount of new, behaviorally relevant information. We find that beta power decreases more strongly when the correct choice of a motor effector in the serial reaction time task (i.e., which finger to move) cannot be predicted before the Go cue, as in the random sequence condition (in *patients P2*, *P3*, and *P5*). This could imply that the observed beta decrease in the NAcc is particularly pronounced when new information relevant for an imminent movement becomes available (note, however, that our task design does not dissociate effects of predictability from reaction time effects between conditions).

### Behavioral Functions of the NAcc

In light of Brittain and Brown's (2014) proposal that beta desynchronization facilitates coding across smaller-scale networks (“liberating” circuits), the beta power decrease observed in our study may, in principle, facilitate integration of afferents to the NAcc as assumed in an influential theory of NAcc function. According to this theory (Floresco 2015; Goto and Grace 2008; Grace 2000), the NAcc influences actions by integrating behaviorally relevant information, e.g., related to context, space, conflict, or affect, from other regions including the hippocampus, prefrontal cortex, and amygdala. While Goto and Grace's emphasis of NAcc functions in action selection has inspired much research, particularly in rodents (e.g., Calhoon and O'Donnell 2013; Gruber et al. 2009; for a review see Floresco 2015), human research on NAcc function has tended to focus on its role in outcome evaluation (and on an indirect influence on future action selection via an evaluation of past action outcomes; e.g., Pessiglione et al. 2006). This focus is also evident across electrophysiological studies of the human NAcc (Cohen et al. 2009a, 2009b, 2009c; Lega et al. 2011; Stenner et al. 2015b; but see Patel et al. 2012; Stenner et al. 2015a). Here, we extend this view of the human NAcc by showing that a signal that indexes preparation for imminent movement in the motor system is also present in the NAcc. We propose that this perimovement beta-ERD may reflect a direct, “online” involvement in preparing for imminent action, in addition to the NAcc's well-studied putative “off-line” role in evaluating past consequences to guide future actions. An “online” involvement of the NAcc in action preparation has been considered before (Pennartz et al. 2009), based on LFP signals in the NAcc of rats before error correction (van der Meer and Redish 2009a, 2009b).

### Differences Between the Two Tasks

Despite differences in the actions required in each of the two tasks, signals with similar spectro-temporal characteristics were observed, resembling well-known patterns in the motor system during preparation for imminent action. In sensorimotor cortex, a decrease of beta power is observed across movements involving different body parts (e.g., Crone et al. 1998) and executed under different conditions, including externally (e.g., Toma et al. 2010) or self-paced (e.g., Pfurtscheller et al. 2003) movements or actions that result from deliberate decision making (e.g., Donner et al. 2009). This ubiquity across motor actions supports the idea of a fundamental, context-independent, behavioral function of sensorimotor beta oscillations (e.g., Brittain and Brown 2014). Our finding of a perimovement signal with spectral and temporal characteristics in the NAcc similar to those typically observed over sensorimotor cortex and in the subthalamic nucleus supports the idea that this region is involved in a cortico-subcortical beta network dealing with aspects of imminent action.

Similar spectro-temporal dynamics across tasks in our study, despite differences in the actions required in each task, also suggest an involvement of these signals in online processes for imminent action in general, beyond specific contexts of explicit reward. However, our findings do not exclude the possibility that these processes may vary with changing task requirements. Indeed, we do not exclude the possibility that, under some circumstances, these perimovement processes may be related to affectively relevant aspects of behavior, e.g., to expected values of potential outcomes.

While the frequency range of the observed perimovement power changes, as well as their direction and their close temporal association with movements, were similar, we found differences in their amplitude and duration between the two tasks. Specifically, peak *t*-values for the perimovement alpha/beta modulation were larger for the value-based decision making task compared with the serial reaction time task (see color scaling in Fig. 2*B* vs. Fig. 3*B*). In principle, this could reflect differences in the time intervals used to compute baseline power in the two tasks. In the serial reaction time task, patients prepared and executed a motor response during most of the time over which baseline power was computed. Perimovement deviations from this baseline are therefore expected to be relatively small compared with the value-based decision making task, where baseline periods included parts of the intertrial interval as well as longer pre- and postmovement periods.

Second, given the short reaction times and intertrial intervals in the serial reaction time task, we cannot exclude the possibility of a temporal overlap of signals from consecutive trials in that task. For example, it is possible that a postmovement increase in the power of alpha/beta oscillations, as observed in *patients P1*, *P2*, *P3* and *P5*, might overlap with the premovement alpha/beta decrease during preparation of the subsequent response in these patients. In this case, the former would be expected to partially mask the latter. Because of its long, variable intertrial interval and long response times, there is no such overlap between signals from consecutive trials in the decision making task, where we observe a pattern of perimovement alpha/beta modulation similar to that in the serial reaction time task.

Differences in the duration of the alpha/beta modulation between the two tasks likely reflect the fact that key presses in the serial reaction time task were instructed and speeded while responses in the value-based decision making paradigm were self-paced. At the sensorimotor cortical level, speeded instructed finger movements, performed at a rate similar to that in our serial reaction time task, are accompanied by a transient decrease in the power of alpha/beta oscillations that lasts a few hundred milliseconds (Toma et al. 2010). Self-paced finger movements, on the other hand, result in a longer-lasting beta power decrease on the order of more than a second (Pfurtscheller et al. 2003).

### Laterality of Observed Perimovement Beta Power Decrease

We found no evidence of a laterality effect of the beta power decrease in DBS channels in our study (contra- vs. ipsilateral to the hand used for movement execution in the decision making task). In sensorimotor cortex, a perimovement beta-ERD occurs over both hemispheres, and its hemispheric lateralization relative to the moved body side is less pronounced than that of the subsequent postmovement beta rebound or the movement-induced gamma enhancement (Jurkiewicz et al. 2006; Muthukumaraswamy et al. 2013). Similarly, the perimovement beta-ERD in the subthalamic nucleus is bilateral (Alegre et al. 2005). In the serial reaction time task in our study, in which all patients responded with their right hand, we found a similar pattern of a beta power decrease in DBS channels of both hemispheres in *patients P1*, *P2*, and *P3* (Fig. 2*B*), albeit only one side survived statistical testing (contralateral to movement in *patients P1* and *P3* and ipsilateral to movement in *patient P2*). This implies a bilateral distribution similar to that in the subthalamic nucleus and over sensorimotor cortex, with no clear evidence of a lateralization in the NAcc with respect to which side of the body is moved.

### Limitations

Our study is limited by constraints of human invasive electrophysiology, specifically by the clinical diagnoses and pharmacotherapy of participants, and we have previously discussed in detail the limitations of such research (Stenner et al. 2015a, 2015b). All patients were treated for pharmacoresistant partial epilepsy. However, etiology, pharmacotherapy, and associated pharmacodynamics varied across patients (Table 1). Consistency of observations across different patient groups has been taken as evidence that these observations relate to general, physiological, rather than disease-specific conditions (e.g., Singh et al. 2011). Similarly, major disease- or treatment-specific effects in our study seem unlikely, given the consistency of the observed beta-ERD across patients with different etiologies and anticonvulsant treatments, although such effects cannot be fully excluded. Furthermore, in all patients, clinical video-EEG monitoring documented a cortical epileptogenic zone (the clinical rationale for NAcc DBS is a suppression of seizure propagation, not of an epileptogenic focus). During recordings patients were constantly under supervision, and no seizure was observed in any patient.

A similar decrease of the power of alpha and beta oscillations as observed for ventral contacts placed in the NAcc was found for the more dorsal DBS contacts located at least partly outside of the NAcc. Future research will have to address the extent to which more dorsal and more lateral regions of the striatum show a beta desynchronization related to movement.

### Conclusions

Preparing for movement is typically accompanied by a decrease of beta power in sensorimotor cortex and the subthalamic nucleus. Our observation of a perimovement beta-ERD in DBS channels in the human NAcc similar to that typically observed in the motor system suggests a direct involvement of the human NAcc in action preparation.

## GRANTS

M.-P. Stenner was supported by Deutsche Forschungsgemeinschaft scholarship STE 2091/1-2. T. Zaehle, J. Voges, H.-J. Heinze, and M.-P. Stenner received funding from Deutsche Forschungsgemeinschaft Sonderforschungsbereich Grant SFB-779 TPA2 and TPA3. R. J. Dolan is supported by a Wellcome Trust Investigator Award (098362/Z/12/Z).

## DISCLOSURES

No conflicts of interest, financial or otherwise, are declared by the author(s).

## AUTHOR CONTRIBUTIONS

M.-P.S., S.D., and R.B.R. conception and design of research; M.-P.S., S.D., and T.Z. performed experiments; M.-P.S., S.D., R.B.R., and J.K. analyzed data; M.-P.S. and S.D. interpreted results of experiments; M.-P.S. prepared figures; M.-P.S. drafted manuscript; M.-P.S., S.D., R.B.R., R.J.D., and M.A.S. edited and revised manuscript; M.-P.S., S.D., R.B.R., T.Z., F.C.S., J.K., J.V., H.-J.H., R.J.D., and M.A.S. approved final version of manuscript.
